# Complete genome sequences of *Salmonella enterica* serovars from eggshell rinsates in Philippine layer farms

**DOI:** 10.1128/mra.00358-26

**Published:** 2026-06-11

**Authors:** Michael Joseph M. Nagpala, Jonah Feliza B. Mora, Rance Derrick N. Pavon, Andrew D. Montecillo, Homer D. Pantua, Windell L. Rivera

**Affiliations:** 1 Pathogen-Host-Environment Interactions Research Laboratory, Institute of Biology, College of Science, University of the Philippines Diliman 172582, Quezon City, Philippines; 2 Microbiology Division, Institute of Biological Sciences, College of Arts and Sciences, University of the Philippines Los Baños 482308, Los Baños, Philippines; 3 BioAssets Corporation, UPLB Science and Technology Park, Los Baños, Philippines; Nanchang University, Nanchang, Jiangxi, China

**Keywords:** *Salmonella* serovars, layer farms, complete genome

## Abstract

We report the complete circular genomes of four *Salmonella enterica* serovars isolated from eggshell rinsates from various layer farms in the Philippines. Genome assembly was performed using a hybrid approach combining Oxford Nanopore long reads and Illumina short reads.

## ANNOUNCEMENT

The transmission of *Salmonella enterica* through eggs remains a significant public health concern (1). *S. enterica* can persist on eggshells or within eggs for extended periods, allowing contamination at multiple points along the egg distribution chain, from farms to retail and household environments (2). Here, we report the first complete genomes of four *S*. *enterica* serovars isolated from eggshell rinsates collected directly from layer farms in the Philippines, providing a valuable reference for investigating the prevalence of *S. enterica* across the farm-to-fork continuum.

The egg samples were collected from three different farms in San Jose, Batangas, Philippines, following local regulations and institutional guidelines. Pre-enrichment was performed as previously described (3), with minor modifications: six eggs were collected directly from the egg belt on the day of lay and immersed in 250 mL buffered peptone water for 10 min. Isolation and detection of *S. enterica* were conducted as described (4). Confirmed *S. enterica* isolates were grown in tryptic soy broth at 37°C for 24 h. Cells (15 mL, OD_600_ = 1.0) were washed with phosphate-buffered saline, resuspended in 500 μL of Zymo DNA/RNA Shield (Zymo Research), and shipped to Plasmidsaurus for hybrid sequencing. DNA was extracted using *Quick*-DNA Fungal/Bacterial Miniprep Kit (Zymo Research), and libraries were prepared with Rapid Barcoding Kit SQK-RBK114.96 (Oxford Nanopore Technologies) using unsheared, non-size-selected DNA and the Illumina DNA Prep Kit (20060059; Illumina). Long-read sequencing was performed on a PromethION P24 (R10.4.1 flow cell) with basecalling via ont-dorado-for-promethion v7.4.12, while 150 bp paired-end short reads were generated on an Illumina NextSeq 2000.

Long reads were filtered with Chopper v0.10.0b (5) using minimum quality 10 and length 1,000, then subsampled into four 5 Mbp-targeted subsets using Autocycler v0.5.2 (6), assembled with Canu v2.3 (7), Flye v2.9.6-b1802 (8), Miniasm v0.3-r179 (9), Plassembler v1.8.0 (10), and Raven v1.8.3 (11). Assemblies were used to construct a compacted de Bruijn graph using “autocycler compress” and pairwise contig distances were calculated via “autocycler cluster” using shared graph paths, followed by UPGMA clustering. Low-confidence clusters were excluded, while “autocycler trim” and “autocycler resolve” removed overlaps and corrected repeats within clusters, producing single consensus contigs per cluster. All consensus contigs were merged using “autocycler combine” to generate final assembly. Illumina reads were quality-checked with FastQC v0.12.1 (12), trimmed with fastp v1.0.1 (13), and used to refine the long-read assembly via Polypolish v0.6.1 (14). The origins of chromosome and plasmids were determined using OriV-Finder (15), and the replicons were rotated using seqkit “rotate” command (16). Annotation was performed using the NCBI Prokaryotic Genome Annotation Pipeline v6.10 (17), and serotyping with SeqSero2 v1.3.1 (18). The antimicrobial resistance genes (ARGs) were annotated using CARD 4.0.1-RGI 6.0.5. (19). Default parameters were used unless stated otherwise.

Table 1 summarizes the read and assembly statistics, serotypes, plasmids, and ARGs of the isolates. Chromosomal resistance was limited to *aac(6′)-laa,* whereas E3Era1 and F4Erc1 harbored plasmids encoding *qnrB5*. Compared with isolates from chicken meat (4), the eggshell isolates exhibited lower ARG diversity, likely reflecting less antimicrobial use in layers than in broilers (20).

**TABLE 1:** Sequence reads, genome assembly statistics, plasmids, annotation, serotypes, and antimicrobial resistance genes (ARGs) of *Salmonella enterica* isolates from egg rinsates collected from layer farms in the Philippines

Parameters	Isolates			
	A7Era1	C1Erc4	E3Era1	F4Erc1
ONT statistics				
*N_50_* (bp)	8,970	10,925	8,973	9,595
Mean read length	5,732.8	6,744.3	5,706.7	5,980.6
Number of reads	110,364	108,100	113,356	75,508
Coverage SRA acc. no.:	136× SRR37503345	144× SRR37503343	136× SRR37503341	96× SRR37503339
Illumina statistics				
Number of reads	8,126,323	8,649,643	15,224,943	13,157,756
Coverage	516×	504×	939×	821×
SRA acc. no.:	SRR37503344	SRR37503342	SRR37503340	SRR37503338
Genome				
Chromosome				
Size in bp:	4,715,744	5,059,352	4,817,546	4,773,345
%GC	52.12	52.11	52.23	52.26
GenBank acc. no.:	JBVVYN010000001.1	JBVVYM010000001.1	JBVVYL010000001.1	JBVVYK010000001.1
Plasmids Size in bp:	p1_A7Era1 4,390	p1_C1Erc4 98,766	p1_E3Era1 7,032	p1_F4Erc1 7,032
GenBank acc. no.:	JBVVYN010000002.1 p2_A7Era1 4,096 JBVVYN010000003.1 p3_A7Era1 3,428 JBVVYN010000004.1 p4_A7Era1 2,160 JBVVYN010000005.1	JBVVYM010000002.1	JBVVYL010000002.1 p2_E3Era1 4,885 JBVVYL010000003.1 p3_E3Era1 4,390 JBVVYL010000004.1 p4_E3Era1 3,428 JBVVYL010000005.1 p5_E3Era1 2,953 JBVVYL010000006.1 p6_E3Era1 2,819 JBVVYL010000007.1 p7_E3Era1 2,495 JBVVYL010000008.1 p8_E3Era1 2,160 JBVVYL010000009.1 p9_E3Era1 1,993 JBVVYL010000010.1	JBVVYK010000002.1 p2_F4Erc1 5,450 JBVVYK010000003.1 p3_F4Erc1 3,429 JBVVYK010000004.1 p4_F4Erc1 3,312 JBVVYK010000005.1 p5_F4Erc1 2,984 JBVVYK010000006.1 p6_F4Erc1 2,965 JBVVYK010000007.1 p7_F4Erc1 2,883 JBVVYK010000008.1 p8_F4Erc1 2,637 JBVVYK010000009.1 p9_F4Erc1 1,917 JBVVYK010000010.1 p10_F4Erc1 1,316 JBVVYK010000011.1
Annotation				
CDS	4,484	4,955	4,611	4,588
rRNAs	22	22	22	22
tRNAs	85	89	87	84
ncRNAs	13	13	13	13
Serotype prediction	16:l,v:-	Weltevreden	47:z4,z23:-	Braenderup
ARGs				
Chromosome	*aac(6’)-laa*	*aac(6’)-laa*	*aac(6’)-laa*	*aac(6’)-laa*
Plasmids			p6_E3Era1: *qnrB5*	p5_F4Erc1: *qnrB5*

## Data Availability

The raw sequencing data and complete genome sequences have been deposited in GenBank under the accession numbers listed in Table 1. The genomes are associated with BioProject PRJNA1431981.
